# Independent Risk Factors and Associated Comorbid Conditions Affecting Intermittent Hypoxia in 569 Patients Diagnosed with OSA

**DOI:** 10.3390/jcm15020627

**Published:** 2026-01-13

**Authors:** Ilker Yilmam, Sureyya Temelli, Ozge Hacer Eker, Osman Nuri Hatipoglu

**Affiliations:** 1 Department of Pulmonary Medicine, Faculty of Medicine, Trakya University, 22030 Edirne, Turkey; ozge.hacer.eker@gmail.com (O.H.E.); nhatip@gmail.com (O.N.H.); 2 Department of Econometrics, Trakya University, 22030 Edirne, Turkey; sureyyadal@trakya.edu.tr

**Keywords:** obstructive sleep apnea, intermittent hypoxia, obesity

## Abstract

**Background/Objectives**: Obstructive sleep apnea (OSA) is characterized by recurrent episodes of complete or partial upper airway collapse during sleep, leading to apnea or hypopnea and recurrent oxygen desaturation. Intermittent hypoxia (IH) and sleep fragmentation have been proposed as key mechanisms contributing to the adverse cardiovascular consequences observed in OSA. The present study aimed to identify clinical variables independently associated with IH in patients with OSA and to examine their relationships with common comorbid conditions. **Methods**: This retrospective study included 569 adult patients diagnosed with obstructive sleep apnea (OSA) by overnight polysomnography (apnea–hypopnea index [AHI] ≥ 5 events/hour) between February 2020 and January 2025 at the Sleep Laboratory of Trakya University Hospital. Demographic characteristics, body mass index (BMI), AHI values, comorbid medical conditions, average nocturnal oxygen saturation, and the duration of intermittent hypoxia (time below 90% SpO_2_ [T90]) were retrieved from the laboratory database. Normality of distribution was assessed using the Kolmogorov–Smirnov test. Group differences were evaluated using the Mann–Whitney U test and the Kruskal–Wallis test with Dunn–Bonferroni post hoc analysis. Correlations were examined using Spearman’s correlation analysis, and variables independently associated with average nocturnal oxygen saturation and intermittent T90 were assessed using multivariable linear regression analysis. **Results**: The presence of hypertension, diabetes mellitus, and comorbid conditions was associated with significant differences in T90 among patients with OSA. T90 also differed significantly across AHI severity grades. Significant negative correlations were observed between nocturnal oxygen saturation and BMI, hypertension, diabetes, comorbidities, and age. Nocturnal oxygen saturation values likewise differed significantly across BMI-defined obesity groups. In the multivariable regression analysis, BMI, AHI, and age were independently associated with lower nocturnal oxygen saturation and longer T90. **Conclusions**: This study provides important insight into the complex relationships among OSA severity, patient demographics, comorbidities, and intermittent hypoxia. In multivariable analysis, BMI, AHI, and age showed independent associations with reduced nocturnal oxygen saturation and prolonged T90. These findings highlight the importance of a multidimensional clinical assessment in OSA and support the use of intermittent hypoxia metrics as additional indicators of disease burden and potential clinical impact.

## 1. Introduction

Obstructive sleep apnea (OSA) is a common sleep disorder characterized by recurrent episodes of upper airway collapse during sleep, leading to intermittent hypoxia and sleep fragmentation. OSA is the most common sleep-related breathing disorder and predominantly affects older men but can also occur in women and children [1]. The incidence of OSA rises postmenopause; thus, rates in postmenopausal individuals are similar to those observed in men. Untreated OSA has many potential consequences, including excessive daytime sleepiness, impaired daytime function, metabolic dysfunction, and an increased risk of cardiovascular disease and mortality.

An estimated 1 billion adults aged 30–69 worldwide experience OSA. A growing body of evidence suggests that OSA contributes to diabetes, hypertension, stroke, ocular complications, cognitive impairment, and ischemic heart disease due to recurrent episodes of oxygen desaturation and reoxygenation [2].

Traditional measures of OSA severity, such as the apnea–hypopnea index (AHI) and oxygen desaturation index (ODI), are widely used to assess OSA severity and guide therapeutic decision-making. However, it is widely accepted that the AHI and ODI have significant limitations, and new physiologically validated measurements are needed to better capture OSA severity and characterize its physiological consequences, particularly the severity of chronic intermittent hypoxia (IH) following respiratory events [3].

This study was conducted to investigate how mean oxygen saturation levels and time below 90% SpO_2_ during sleep (T90) are influenced by OSA severity (AHI), age, obesity (body mass index [BMI]), and the presence of comorbidities such as hypertension, diabetes, and coronary artery disease. Additionally, potential sex and age differences in mean oxygen saturation and IH values among OSA patients were explored.

## 2. Materials and Methods

### 2.1. Study Population

Data from patients aged 18 or older diagnosed with OSA between February 2020 and January 2025 at the Sleep Laboratory of Trakya University Hospital were retrospectively evaluated. Five hundred sixty-nine patients with accessible data were included. Age, sex, BMI, weight, height, AHI values, comorbid conditions (hypertension, diabetes, coronary artery disease, thyroid disorders, arrhythmias, psychiatric disorders, etc.), average sleep oxygen saturation, and T90 were obtained from the sleep laboratory database.

### 2.2. Exclusion Criteria

Patients diagnosed with central sleep apnea on polysomnography (PSG), those using positive airway pressure (PAP) therapy, and patients with conditions that may cause hypoxia—such as interstitial lung disease, advanced chronic obstructive pulmonary disease, and heart failure—were excluded from the study.

OSA was classified as mild (AHI ≥ 5 and <15 events per hour), moderate (AHI ≥ 15 and <30 events per hour), or severe (AHI ≥ 30 events per hour). To account for potential age-related differences in OSA presentation and severity, patients were divided into three age groups: ≤29 years, 30–59 years, and ≥60 years.

Furthermore, given the important role of body weight in OSA, the patients were divided into five groups according to BMI: BMI < 25, normal; BMI 25–29.9, overweight; BMI 30–34.9, obese class I; BMI 35–39.9, obese class II; and BMI ≥ 40, obese class III.

### 2.3. Polysomnographic (PSG) Examinations

All patients undergoing polysomnographic examinations provided pre-procedural consent.

Sleep recordings were performed by applying 3-channel electroencephalography (F4-M1, C4-M1 and O2-M1), 2-channel electrooculography and submental electromyography with electrodes placed according to the international 10 to 20 system. Cardiorespiratory recording was performed by monitoring snoring, oral and nasal airflow (by thermistor and nasal cannula), thoracic–abdominal movements, position, and pulse oximetry (Alice-6, Philips Respironics, Murrysville, PA, USA). Periodic leg movements were also recorded with synchronous tibialis anterior electromyography and video recording.

A certified sleep physician manually scored all recordings. Sleep-related abnormal respiratory events were scored according to the American Academy of Sleep Medicine (AASM 2017) criteria [4]. Apnea was defined as the complete cessation of oral and nasal airflow ≥10 s. The criteria for hypopnea scoring were accepted as a decrease in airflow lasting ≥10 s leading to arousal or oxygen desaturation (represented by a decrease in oxygen saturation greater than 3%).

T90 was defined as the percentage of total sleep time (TST) spent with SpO_2_ < 90% during sleep.

### 2.4. Ethical Approval

This study was approved by Trakya University School of Medical science ethical committee (The ethical code TUTF-GOBAEK 2025/386, no: 17/11, Date of approval: 22 September 2025).

### 2.5. Statistical Analysis

Patients diagnosed with OSA were evaluated according to their medical conditions. Data analysis was conducted using IBM SPSS Statistics 26.0 software (IBM Corp., Armonk, NY, USA). A significance level of *p* < 0.05 was employed for all statistical analyses. Average saturation and T90 are expressed as the median and interquartile range for each medical condition. Medical conditions, including sex, age, degree of severity (AHI), BMI, hypertension, coronary artery disease, diabetes, and comorbidities are presented as frequencies and percentages. Medical conditions were analyzed by the Chi-square and Fisher’s exact tests. The Kolmogorov–Smirnov test, along with measures of kurtosis and skewness, was employed to assess the normality of the data distribution. When the assumption of normality was violated, the differences between groups were examined using the Mann–Whitney U and Kruskal–Wallis H (K-W-H) tests. After obtaining significant findings from the K-W-H test, post hoc pairwise comparisons were performed using Dunn’s test with Bonferroni correction to control for type I error inflation due to multiple testing. Then, correlations between variables were examined using Spearman’s correlation analysis, given the data distribution. Missing data were not imputed; cases with missing values in any of the variables included in the regression model were excluded using listwise deletion. A multiple regression analysis was performed to examine independent associations among the variables. Collinearity diagnostics indicated no significant multicollinearity (all VIF < 10; condition index < 30). Standardized regression coefficients from the multiple regression analysis were used to compare the effects of the variables on average saturation levels and the T90.

## 3. Results

A total of 569 patients with OSA were included in the analysis. Most of the patients were male (73.64%), aged 30–59 years (73.11%), and had comorbidities (65.91%). With respect to OSA severity, 24.6% of the patients had mild OSA, 22.7% had moderate OSA, and 52.7% had severe OSA. The distribution of demographic and clinical characteristics across AHI severity categories is presented in Table 1. Significant differences in AHI severity were observed across sex, age, and BMI groups (*p* < 0.001). However, there were no significant differences in AHI severity with respect to hypertension, coronary artery disease, diabetes mellitus, or the presence of comorbidities (*p* = 0.438, *p* = 0.754, *p* = 0.384, and *p* = 0.295, respectively).

Average saturation, T90, and AHI were not normally distributed (*p* < 0.001) (Table 2). Thus, nonparametric tests were conducted on the average saturation and T90 indicators, which are critical for monitoring patients with OSA.

**Figure 1:**
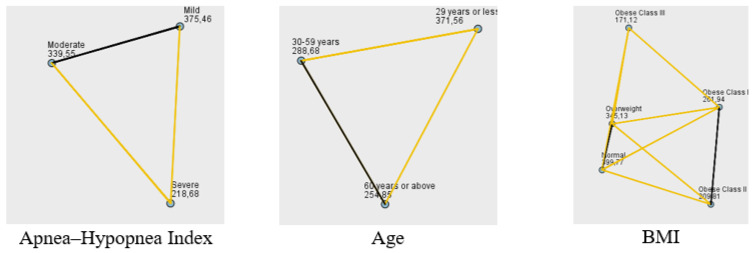
Pairwise Comparisons of Average Saturation. **Note:** Mean ranks are presented. Black lines indicate significant pairwise differences. Formatting limitations of SPSS prevented full typographical customization. Label overlaps do not affect interpretation.

Various statistical methods were employed to analyze the relationships between average saturation values and patient characteristics, including the K-W-H test, Dunn’s test with Bonferroni correction, and the Mann–Whitney U test. Our findings provide valuable insights into the complex interplay between OSA severity, patient demographics, comorbidities, and average oxygen saturation and IH during sleep. Average saturation values were evaluated according to medical conditions. The K-W-H test was used to determine whether average saturation values differed across AHI levels. The analysis revealed a significant difference (*p* < 0.001) (Table 3). Dunn’s test with Bonferroni correction was applied for post hoc analyses. Significant differences (*p* < 0.001) were found between the moderate and severe group and between the severe and mild group (Figure 1). In addition to AHI levels, we investigated the impact of age on average saturation levels in OSA patients. The average saturation levels varied among OSA patients across different age categories (*p* = 0.004) (Table 3). In the pairwise comparisons, a notable difference was observed between individuals ≤29 years and those ≥60 years, as well as between individuals ≤29 years and those aged 30–59 (Figure 1).

After establishing the influence of age, we next explored the relationship between obesity and nocturnal oxygen saturation. Average saturation values differed significantly across BMI-based obesity groups (*p* < 0.001, Kruskal–Wallis test) (Table 3). Pairwise post hoc comparisons showed that patients in the obese class III group had significantly lower saturation values than those in the obese class I, overweight, and normal-weight groups. In addition, obese class II patients had significantly lower saturation levels than overweight and normal-weight patients, and obese class I patients also differed significantly from overweight and normal-weight individuals (Figure 1).

We then examined the potential impact of common comorbidities on nocturnal oxygen saturation. Average saturation values were significantly lower in patients with hypertension, diabetes mellitus, and comorbidities compared with those without these disorders (*p* <0.001, *p* = 0.011, and *p* = 0.003, respectively; Mann–Whitney U test). By contrast, no significant differences in saturation were observed according to sex or the presence of coronary artery disease (*p* = 0.714 and *p* = 0.270, respectively) (Table 3).

Building on these findings, we further examined the relationship T90 and clinical characteristics. T90 increased progressively with AHI severity and differed significantly across AHI categories (*p* < 0.001, Kruskal–Wallis test). T90 also differed significantly across BMI groups (*p* < 0.001), with the longest hypoxic burden observed in patients with class II and class III obesity. With respect to comorbid medical conditions, patients with hypertension, diabetes mellitus, and comorbidities had significantly longer T90 compared with those without these conditions (*p* = 0.003, *p* = 0.005, and *p* = 0.007, respectively; Mann–Whitney U test). In contrast, T90 did not differ significantly according to the presence of coronary artery disease (*p* = 0.143) or sex (*p* = 0.315) (Table 4). Finally, T90 differed significantly across age categories, with higher values in older age groups (*p* = 0.008, Kruskal–Wallis test).

**Figure 2:**
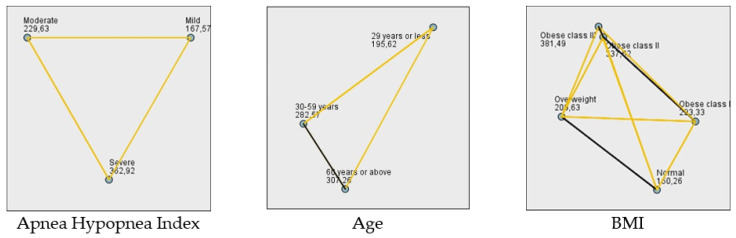
Pairwise Comparisons of IH. **Note:** Mean ranks are presented. Black lines indicate significant pairwise differences. Formatting limitations of SPSS prevented full typographical customization. Label overlaps do not affect interpretation.

T90 differed significantly across AHI severity categories (*p* < 0.001, Kruskal–Wallis test) (Table 4; Figure 2). Patients with severe OSA exhibited the longest hypoxic burden, followed by those with moderate and mild disease. T90 also differed significantly across BMI categories (*p* < 0.001), with a progressive increase in hypoxic burden from normal weight to obese class III. Post hoc pairwise comparisons demonstrated significant differences between most BMI categories, particularly involving class II and class III obesity. These findings indicate that increasing body weight is associated with a clear and clinically meaningful prolongation of nocturnal hypoxia. We further evaluated the influence of age, and T90 was also found to differ significantly among age groups (*p* = 0.008), with higher values generally observed in older individuals. The K-W-H test revealed a significant difference in IH values across different age groups (*p* = 0.008; Table 4). The post hoc analysis identified that this difference is specifically between the ≤29- and 30–59-year age groups and the ≤29- and ≥60-year age groups (Figure 2).

To provide an overview of the associations among clinical variables, we initially performed a correlation analysis between intermittent hypoxia (IH) parameters and patient characteristics. Average nocturnal oxygen saturation showed significant negative correlations with BMI, hypertension, diabetes mellitus, comorbidities, and age, while T90 was positively correlated with the same variables. No significant correlations were observed for coronary artery disease or sex (Table 5). However, because correlation analysis does not account for interactions among variables, greater emphasis was placed on multivariable regression analysis to identify independent predictors of IH.

Finally, we conducted a multivariable regression analysis to identify which parameters were independently associated with intermittent hypoxia measures. Collinearity diagnostics indicated no significant multicollinearity (all VIF < 10; condition index < 30). Standardized regression coefficients (β) were used to assess and compare the relative impact of each variable on average oxygen saturation and IH levels. Table 6 presents the associations between average saturation, hypoxia 90%, and the examined medical conditions. In the multivariable regression analysis, BMI (β = −0.365, *p* < 0.001), AHI (β = −0.273, *p* < 0.001), and age (β = −0.100, *p* = 0.016) were independently associated with lower average oxygen saturation. Among these, BMI had the strongest negative effect, followed by AHI and age. Other variables, including diabetes, hypertension, coronary artery disease, comorbidities, and sex, did not reach significance. Furthermore, BMI (β = 0.350, *p* < 0.001), AHI (β = 0.264, *p* < 0.001), and age (β = 0.134, *p* = 0.001) were independently associated with higher IH levels. Among these, BMI showed the strongest effect, followed by AHI and age. Other variables, including diabetes, hypertension, coronary artery disease, comorbidities, and sex, did not reach significance.

## 4. Discussion

This study provides valuable insights into the complex relationships among OSA severity, patient demographics, comorbidities, and sleep oxygen saturation. The findings demonstrate significant associations between AHI severity, age, BMI, and various comorbidities with average oxygen saturation and nocturnal hypoxia values. These results underscore the importance of considering multiple factors when assessing OSA patients and highlight the potential utility of oxygen saturation metrics as additional indicators of disease severity and impact.

In the multivariate regression analysis, BMI, AHI, and age were found to be independently associated with nocturnal average oxygen saturation and T90. As BMI, AHI, and age increased, patients exhibited greater hypoxic burden. Although they were statistically significant in univariate analyses, the loss of statistical significance of comorbidities in the multivariate regression model is a striking finding of our study. We clarified that the effects of hypertension, diabetes mellitus, and comorbidities on nocturnal IH were largely associated with BMI and age, rather than being independent relationships. After BMI, age, and AHI were included in the multivariate model, comorbidities no longer remained significant predictive factors, indicating that obesity, advanced age, and OSA severity account for most of the variance in hypoxic load.

Age and sex are two factors that play a role in OSA prevalence; men have a two- to three-fold higher risk of OSA when compared with women, and the proportion of individuals >60 years of age with an AHI ≥ 15 is approximately 1.7 times higher than that of middle-aged individuals [5,6]. The sex difference in risk has been attributed to the influence of sex hormones and differences in fat distribution, smoking, alcohol consumption, and craniofacial structure [7,8]. In our study, consistent with the literature, males predominated (73.64%). This may reflect both the higher prevalence of OSA in men and greater referral of working-age men to sleep clinics. The majority of our cohort consisted of individuals aged 30–59 years (73.11%).

The prevalence of OSA appears to be increasing, which may be associated with rising obesity rates and improved OSA detection [9,10]. Obesity is one of the strongest established risk factors for OSA [11,12,13]. In our sample, 61.25% of patients were obese and 27.94% were overweight. As BMI increased, average sleep oxygen saturation decreased and T90 increased significantly, supporting the close relationship between excess body weight and OSA-related hypoxia.

Cardiovascular diseases (CVDs) and traditional CVD risk factors are common in adults diagnosed with OSA. Obesity, insulin resistance, diabetes mellitus, and hyperlipidemia, together with OSA, may have synergistic and/or additive effects on the development of CVD [14,15]. Given the higher prevalence of OSA in CVD cohorts than in the general population, a bidirectional interaction has been proposed.

In our study, 65.91% of OSA patients had comorbid conditions. Hypertension (37.26%) was the most common comorbidity, followed by diabetes mellitus (21.62%) and coronary artery disease (9.14%). In univariate analyses, hypertension, diabetes, and comorbidities were associated with both lower mean nocturnal oxygen saturation and longer T90. Prior studies suggest that IH and sleep fragmentation may contribute to metabolic dysregulation [16]. However, in our multivariable regression model, these comorbidities were no longer significant predictors of nocturnal hypoxia once BMI and AHI were included. This finding suggests that the observed associations between comorbidities and IH may be largely mediated through obesity and OSA severity.

This study has several limitations. First, its retrospective design precludes any causal inference. Second, all data were obtained from a single tertiary sleep center, which may limit the generalizability of the findings. Third, although key clinical variables were included in the analyses, the potential influence of unmeasured or residual confounders (e.g., lifestyle factors, medication use, and the duration or control of comorbidities) cannot be excluded. Finally, the cross-sectional nature of the analysis does not allow conclusions regarding the temporal relationship between obesity, OSA severity, comorbidities, and intermittent hypoxia.

## 5. Conclusions

Our findings demonstrate that intermittent hypoxia (IH) is significantly associated with AHI severity, age, BMI, and the presence of comorbid medical conditions in patients with OSA. Higher BMI was strongly associated with a greater hypoxic burden and with more pronounced nocturnal hypoxemia. Chronic IH has also been linked in the prior literature to adverse effects on metabolic regulation and body-weight control; however, the retrospective design of the present study does not allow conclusions regarding the directionality or causality of these relationships. Overall, these results emphasize the importance of considering multiple clinical factors beyond AHI alone when evaluating patients with OSA and support the use of IH-related metrics as complementary indicators of disease severity and potential clinical impact.

## Figures and Tables

**Table 1:** Distribution of Medical Conditions and Their Association with Varying Severities of the Apnea–Hypopnea Index.

	All Patients		Apnea Hypopnea Index						*p*
			Mild		Moderate		Severe		
			140 (24.6%)		129 (22.7%)		300 (52.7%)		
	n	%	n	%	n	%	n	%	
Gender									
Male	419	73.64%	87	62.14%	93	72.09%	239	79.67%	<0.001
Female	150	26.36%	53	37.86%	36	27.91%	61	20.33%	
Age									
29 years or less	24	4.22%	12	8.57%	4	3.10%	8	2.67%	0.042 *
30–59 years	416	73.11%	96	68.57%	92	71.32%	228	76.00%	
60 years or above	129	22.67%	32	22.86%	33	25.58%	64	21.33%	
BMI									
Normal	46	8.08%	26	18.57%	9	6.98%	11	3.67%	<0.001
Overweight (BMI 25.0–29.9)	159	27.94%	46	32.86%	43	33.33%	70	23.33%	
Obese class I (BMI 30.0–34.9)	181	31.90%	40	28.57%	41	31.78%	100	33.33%	
Obese class II (BMI 35.0–39.9)	94	16.52%	13	9.29%	20	15.50%	61	20.33%	
Obese class III (BMI > 40)	73	12.83%	12	8.57%	10	7.75%	51	17.00%	
Hypertension									
Yes	212	37.26%	46	32.86%	50	38.76%	116	38.67%	0.438
No	355	62.39%	94	67.14%	77	59.69%	184	61.33%	
Coronary artery disease									
Yes	52	9.14%	12	8.57%	10	7.75%	30	10.00%	0.754
No	515	90.51%	128	91.43%	117	90.70%	270	90.00%	
Diabetes									
Yes	123	21.62%	25	17.86%	27	20.93%	71	23.67%	0.384
No	444	78.03%	115	82.14%	100	77.52%	229	76.33%	
Comorbid Medical Conditions									
Yes	375	65.91%	85	60.71%	86	66.67%	204	68.00%	0.295
No	192	33.74%	55	39.29%	41	31.78%	96	32.00%	

**Note:** Missing data were as follows—BMI: 16 cases (2.81% overall; 2.14% mild, 4.65% moderate, 2.33% severe); hypertension, coronary artery disease, diabetes mellitus and comorbidities: 2 cases each (0.35% overall; 0.00% mild, 1.55% moderate, 0.00% severe). * Significant at *p* < 0.05. BMI: Body mass index.

**Table 2:** Kolmogorov–Smirnov Normality Test Results.

	Kurtosis	Skewness	Statistic	*p*
Average Saturation	5.445	−1.965	0.185	<0.001
T90	0.253	1.123	0.186	<0.001
AHI	−0.714	0.626	0.140	<0.001

**Note:** T90: time below 90% SpO_2_, AHI: Apnea–hypopnea index.

**Table 3:** Distribution of Average Saturation Across Medical Conditions.

	Medical Conditions		Median (IQR)	*n*
Average Saturation	Apnea–Hypopnea Index	Mild		139
		Moderate		129
		Severe		300
		*p* < 0.001 (K-W-H)		
	BMI	Normal	93.50 (2.00)	46
		Overweight	93.00 (3.00)	159
		Obese class I	91.50 (4.00)	180
		Obese class II	90.00 (6.63)	94
		Obese class III	88.50 (7.30)	73
		*p* < 0.001 (K-W-H)		
	Hypertension	Yes	91.00 (4.50)	211
		No	92.00 (4.00)	355
		*p* < 0.001 (M-W-U)		
	Coronary artery disease	Yes	91.00 (5.30)	52
		No	91.50 (4.00)	514
		*p* = 0.270 (M-W-U)		
	Diabetes	Yes	91.00 (4.00)	122
		No	91.50 (4.50)	444
		*p* = 0.011 * (M-W-U)		
	Comorbidities	Yes	91.03 (4.50)	374
		No	92.00 (3.88)	192
		*p* = 0.003 * (M-W-U)		
	Sex	Male	91.50 (4.10)	418
		Female	91.50 (4.50)	150
		*p* = 0.714 (M-W-U)		
	Age	≤29 years	93.75 (4.25)	24
		30–59 years	91.50 (4.50)	415
		≥60 years	91.00 (4.00)	129
		*p* = 0.004 * (K-W-H)		

**Note:** * Significant at the 0.05 level. BMI: Body mass index.

**Table 4:** Distribution of T90 Across Medical Conditions.

	Medical Conditions		Median (IQR)	*n*
T90	Apnea–Hypopnea Index	Mild	1.58 (8.61)	140
		Moderate	5.03 (13.72)	129
		Severe	30.6 (38.03)	299
		*p* < 0.001 (K-W-H)		
	BMI	Normal	1.33 (10.64)	46
		Overweight (BMI 25.0–29.9)	5.28 (20.27)	159
		Obese class I (BMI 30.0–34.9)	15.29 (36.79)	180
		Obese class II (BMI 35.0–39.9)	31.76 (44.52)	94
		Obese class III (BMI > 40)	42.70 (63.73)	73
		*p* < 0.001 (K-W-H)		
	Hypertension	Yes	18.17 (38.36)	211
		No	9.5 (36.53)	355
		*p* = 0.003 * (M-W-U)		
	Coronary artery disease	Yes	22.99 (36.69)	52
		No	11.72 (38.62)	514
		*p* = 0.143 (M-W-U)		
	Diabetes	Yes	19.52 (40.60)	123
		No	11.39 (37.31)	443
		*p* = 0.005 * (M-W-U)		
	Comorbidities	Yes	15.57 (39.20)	374
		No	8.84 (32.48)	192
		*p* = 0.007 * (M-W-U)		
	Sex	Male	12.18 (37.79)	419
		Female	10.68 (40.42)	149
		*p* = 0.315 (M-W-U)		
	Age	≤29 years	2.13 (37.67)	24
		30–59 years	12.28 (38.09)	415
		≥60 years	13.35 (38.04)	129
		*p* = 0.008 * (K-W-H)		

**Note:** * Significant at the 0.05 level. BMI: Body mass index, T90: time below 90% SpO_2_.

**Table 5:** Spearman’s Correlation Coefficients Between Sleep Apnea Markers and Medical Conditions.

Markers	Medical Conditions	*n*	Rho	*p*
Average nocturnal O_2_ Saturation	BMI	552	−0.440 *	<0.001
	Hypertension	566	−0.151 *	<0.001
	Coronary artery disease	566	−0.046	0.270
	Diabetes	566	−0.107 *	0.011
	Comorbidities	566	−0.127 *	0.002
	Sex	568	0.015	0.714
	Age	568	−0.127 *	0.002
Time below 90% SpO_2_ (T90)	BMI	552	0.433 *	<0.001
	Hypertension	566	0.125 *	0.003
	Coronary artery disease	566	0.062	0.144
	Diabetes	566	0.119 *	0.004
	Comorbidities	566	0.114 *	0.007
	Sex	568	−0.042	0.315
	Age	568	0.107 *	0.011

**Note**: * Significant at the 0.05 level. BMI: Body mass index.

**Table 6:** Multivariable Regression Analysis of Factors Associated with Average Oxygen Saturation and time below 90% SpO_2_.

Markers	Medical Conditions	B	β	*p*
Average Saturation $R^{2}=0.267$, p<0.001	BMI	−1.475 *	−0.365 *	<0.001
	Hypertension	−0.191	−0.020	0.664
	Coronary artery disease	0.851	0.053	0.168
	Diabetes	0.608	0.054	0.193
	Comorbidities	−0.328	−0.033	0.477
	Sex	0.400	0.038	0.336
	Age	−0.039 *	−0.100 *	0.016
	AHI	−1.515 *	−0.273 *	<0.001
	Constant	97.123 *	-	<0.001
IH $R^{2}=0.259$, p<0.001	BMI	8.296 *	0.350 *	<0.001
	Hypertension	−1.059	−0.019	0.683
	Coronary artery disease	−1.160	−0.012	0.750
	Diabetes	−2.144	−0.032	0.435
	Comorbidities	3.471	0.060	0.202
	Sex	−1.605	−0.026	0.513
	Age	0.311 *	0.134 *	0.001
	AHI	8.603 *	0.264 *	<0.001
	Constant	−19.436 *	-	<0.001

**Note:** AHI: Apnea–hypopnea index, BMI: Body mass index. * *p* < 0.05.

## Data Availability

Data of the patients included in this study were obtained from the electronic medical record system of The Sleep Laboratory of Trakya University Hospital and are not publicly accessible. De-identified data may be provided by the corresponding author upon reasonable request and with approval from the institutional ethics committee.
